# Evaluation of toxicity of clothianidin (neonicotinoid) and chlorfenapyr (pyrrole) insecticides and cross-resistance to other public health insecticides in *Anopheles arabiensis* from Ethiopia

**DOI:** 10.1186/s12936-019-2685-2

**Published:** 2019-02-22

**Authors:** Kendra Dagg, Seth Irish, Ryan E. Wiegand, Josephat Shililu, Delenasaw Yewhalaw, Louisa A. Messenger

**Affiliations:** 1 Faculty of Infectious Tropical Diseases, London School of Hygiene and Tropical Medicine, London, UK; 2 Entomology Branch, Centers for Disease Control and Prevention, 1600 Clifton Road, Atlanta, GA 30329-4027 USA; 3 President’s Malaria Initiative, Bureau for Global Health, Office of Infectious Disease, United States Agency for International Development, Washington, DC USA; 4 President’s Malaria Initiative Africa Indoor Residual Spraying Project, Abt Associates, Gerji Road, Sami Building 1st Floor, Addis Ababa, Ethiopia; 5 Tropical and Infectious Diseases Research Center, Jimma University, Jimma, Ethiopia; 6 Department of Medical Laboratory Sciences and Pathology, College of Health Sciences, Jimma University, Jimma, Ethiopia; 7 American Society for Microbiology, 1752 N Street, NW, Washington, DC 20036 USA

**Keywords:** *Anopheles arabiensis*, Insecticide resistance, Cross-resistance, Clothianidin, Chlorfenapyr, Indoor residual spraying, Long-lasting insecticidal nets, Ethiopia

## Abstract

**Background:**

Insecticide-based interventions play an integral role in malaria vector control. However, the continued spread of insecticide resistance threatens to undermine progress made thus far and may ultimately lead to operational failure of current control measures. Clothianidin and chlorfenapyr both have unique modes of action and have expanded the number of insecticide classes available to vector control programmes. Prior to field use, it is imperative to establish their toxicity against local mosquito populations and evaluate potential cross-resistance with other chemicals used contemporarily or historically. The aim of this study was to determine the diagnostic doses of clothianidin and chlorfenapyr and their efficacies against *Anopheles arabiensis*, the predominant Ethiopian malaria vector species.

**Methods:**

A range of doses of clothianidin and chlorfenapyr were tested, using modified WHO susceptibility tests and CDC bottle bioassays, respectively, against an Ethiopian susceptible laboratory strain and a wild population of *An. arabiensis* collected from Oromia Region, Ethiopia. Cross-resistance to other public health insecticides: carbamates (bendiocarb and propoxur), organophosphate (malathion) and pyrethroids (deltamethrin and permethrin), was assessed in the same mosquito populations using CDC bottle bioassays.

**Results:**

Complete mosquito mortality was observed with the laboratory strain using the recommended diagnostic doses for clothianidin (2%/filter paper) and chlorfenapyr (100 µg/bottle). The field population was resistant to malathion (83% mortality), capable of surviving 2×, 5× and 10× the diagnostic dose of both deltamethrin and permethrin, but susceptible to bendiocarb and propoxur. The field population of *An. arabiensis* was significantly more susceptible to clothianidin, reaching 100% mortality by day 2 compared to the laboratory strain (100% mortality by day 3). In contrast, the wild population was less susceptible to chlorfenapyr, with the highest mortality of 99% at 72 h using 200 µg/bottle compared to the laboratory colony, which reached complete mortality at 50 µg/bottle by 24 h.

**Conclusions:**

The putative diagnostic doses of clothianidin and chlorfenapyr are appropriate for monitoring resistance in *An. arabiensis* from Ethiopia. The unique modes of action and an absence of cross-resistance render clothianidin and chlorfenapyr potential candidates for inclusion in the National Malaria Control Programme vector control efforts, particularly in areas with high pre-existing or emergent resistance to other insecticide classes.

## Background

In recent years, substantial achievements have been made in global malaria control which has led to an estimated 22% and 29% decrease in malaria incidence and mortality, between 2000 and 2015, respectively [1]. These impressive achievements have largely resulted from the rapid scale-up of diagnosis, treatment and vector control interventions, particularly indoor residual spraying (IRS) and long-lasting insecticidal nets (LLINs). Unfortunately, the expansion of IRS coverage and concomitant mass LLIN distributions have placed high levels of selection pressure on *Anopheles* mosquito populations to evolve resistance to the thirteen insecticides belonging to the four main classes approved for public health use: pyrethroids, carbamates, organophosphates, and organochlorines [2, 3]. Moreover, the rate of decline in malaria case incidence has begun to stall and has even reversed in some regions since 2014 [4]. Insecticide resistance is now a pervasive phenomenon that has been reported in approximately two-thirds of countries with ongoing malaria transmission [1, 3, 5]. In addition, many vector populations are resistant to multiple insecticides from different chemical classes; of the 73 countries that provided monitoring data from 2010 onwards, 50 reported resistance to two or more insecticide classes [1]. The continued spread of resistance could threaten malaria control progress achieved thus far and ultimately lead to operational failure of prevailing control measures [3]. In response, the current recommendations for insecticide resistance management rely on tactical deployment of the active ingredients used for IRS and on LLINs in rotation, combinations (particularly LLINs), mosaics and mixtures [3, 6]. Unfortunately, these management strategies are restricted in their potential effectiveness by the limited choice of available insecticides. The urgent need for new chemicals with novel modes of action has been the impetus driving the evaluation of established agricultural insecticides to control resistant mosquito vector populations [7].

Historically, the chemical industry has not focused on the development of new public health insecticides because of high market uncertainties and low profit margins in comparison to the agricultural sector [8, 9]. However, current efforts are focusing on testing insecticides already available to the agricultural industry as potential public health tools. The ideal insecticide for IRS and LLINs should possess the following properties: intrinsic toxicity, chemical and physical properties that facilitate effective uptake upon contact, long residual efficacy, toxicity to specific mosquito species at low dosages, easy application to the desired substrate, stability, low volatility, and low mammalian toxicity (including to other non-target species). Chlorfenapyr and clothianidin are two such insecticides, belonging to different chemical classes with distinct modes of action.

Chlorfenapyr is a pyrrole class insecticide commonly used against mites and termites, which functions as an oxidative phosphorylation uncoupler. This compound disrupts the proton gradient across mitochondrial membranes, interrupting ATP synthesis and ultimately resulting in death of the organism [10, 11]. A susceptibility survey of 19 pesticides tested against insectary colonies of *Aedes aegypti, Culex quinquefasciatus* and *Anopheles quadrimaculatus* indicated that chlorfenapyr was most effective against *An*. *quadrimaculatus* and least against *Culex quinquefasciatus* [12]; other toxicity screens have reported that chlorfenapyr can have a lethal effect against field populations of the latter species [13]. Furthermore, multiple phase II trials in Tanzania and Benin have highlighted the effectiveness of chlorfenapyr as an adjunct to pyrethroid-treated nets against *Anopheles arabiensis, Anopheles gambiae* sensu stricto (s.s.), and *Culex quinquefasciatus* [14–18] and as a candidate for IRS in Benin [8, 10].

Clothianidin is one of seven insecticides within the neonicotinoid class; it has low mammalian toxicity and is primarily used against piercing–sucking insects of major crops [19, 20]. The basic mode of action is to target the nicotinic acetylcholine receptor (nAChR) in the insect central nervous system [19, 21]. Compared with chlorfenapyr, this class of insecticide has been through less rigorous study in relation to vector control. At a molecular level, each neonicotinoid has been characterized by differential activity against the nAChR protein subunit of *An. gambiae*, suggesting that these compounds may have differential efficacies against target insects [21]. In a toxicity survey examining 25 different synthetic insecticides, clothianidin was among the group of six neonicotinoids inducing the highest mortality levels against *Culex quinquefasciatus* [13]. Toxicity of six neonicotinoids was tested alone and in combination with deltamethrin and the synergist piperonyl butoxide (PBO) against *Ae. aegypti* and *An. gambiae* and all compounds had poor individual efficacies but induced higher levels of insecticidal action when in combination, possibly due to the amelioration of oxidase and/or esterase activity by PBO [22]. Clothianidin, both alone and in mixtures, demonstrated some of the lowest mortality rates among the six compounds. However, it is noteworthy that this study did not evaluate a range of doses for each insecticide, nor monitor mosquito mortality past 24 h; given the slower acting nature of these compounds and that each neonicotinoid can produce its own unique range of sub-lethal effects, further testing is warranted [22].

Both chlorfenapyr and clothianidin have been manufactured into new commercial vector control formulations. Chlorfenapyr is one of two active ingredients in a combination LLIN with alpha-cypermethrin, produced by BASF (Interceptor^®^ G2), which received interim approval from the World Health Organization (WHO) in 2017 [23]. It is also under evaluation as an IRS product (Sylando^®^ 240SC). Clothianidin has been developed by Sumitomo into an IRS formulation (SumiShield^®^ 50WG), which has been pre-qualified by the WHO [24], and Bayer has also incorporated clothianidin in a mixture IRS product with deltamethrin (Fludora™ Fusion WP-SB) [25]. To date, phase II trials of these respective interventions have reported promising results with multiple resistant vector species [8, 10, 14, 17, 22, 26, 27]. Furthermore, a recent phase III trial in India, demonstrated greater reductions in the density of indoor, pyrethroid-resistant *Anopheles culicifacies* with SumiShield^®^ 50WG, compared to Actellic^®^ 300CS (pirimiphos-methyl) [28]. Prospective community-level implementation of such interventions, as part of National Malaria Control Programmes (NMCPs), is first predicated on demonstrable efficacy against local vector populations.

Insecticide resistance is a major public health concern in Ethiopia, where intensive DDT spraying since 1959 and mass LLIN distributions over the past 10–15 years have resulted in highly focal and heterogeneous patterns of insecticide resistance across the country [29]. In 2016, decreased susceptibility to pyrethroids (alpha-cypermethrin, deltamethrin, etofenprox, lambda-cyhalothrin and permethrin) and incipient resistance to bendiocarb, pirimiphos-methyl and propoxur, the three chemicals routinely used for IRS, were detected in multiple regions [29]. The aim of this study was to determine the diagnostic doses of chlorfenapyr and clothianidin, defined as the concentration of insecticide that kills 100% of susceptible mosquitoes within a given time [30], against a susceptible laboratory strain of *An. arabiensis* and screen for resistance in a field-collected multi-resistant *An. arabiensis* population from Oromia region, Ethiopia. Establishment of the diagnostic doses will provide a critical starting point to monitor future resistance and define the suitability of these insecticides for intervention deployment. In addition, improved understanding of the cross-resistance between these novel chemicals and currently used insecticides will aid the NMCP and other stakeholders in making informed choices regarding the most appropriate tools for malaria vector control and insecticide resistance management.

## Methods

### Mosquito strains

Two populations of *An. arabiensis* were used in this study. An insectary-reared strain of *An. arabiensis* (Debre Zeit; DZ) was provided by the Tropical and Infectious Diseases Research Center, Jimma University in Sekoru, Oromia region (7^o^54′50.0″N, 37^o^25′23.6″E). This strain is known to be susceptible to pyrethroid, carbamate, organochlorine and organophosphate insecticides [31]. Wild *An. gambiae* sensu lato (henceforth referred to as *An. arabiensis*) based on PCR data from local entomological surveys conducted between 2012 and 2016) [29, 32] were collected as blood-fed adults from inside houses and animal shelters in Asendabo, Oromia region (7^o^40′31″N, 36^o^52′56″E) during the long rainy season in June–August 2017, and reared at the Jimma University Tropical and Infectious Diseases Research Center. Additional field sampling for chlorfenapyr testing was undertaken in the same area of Asendabo in September 2018.

F_1_ adults were generated using the forced-oviposition method described by Morgan et al. [33]. Blood-fed, field-collected mosquitoes, morphologically identified as *An. arabiensis* [34], were maintained for 4–5 days until gravid. Each fully gravid female was transferred to a 1.5 ml micro-centrifuge tube containing a slightly wet filter paper on top of damp cotton wool and allowed to lay eggs. Eggs were pooled to reduce any bias due to family effects in larval trays for rearing to the adult stage. Previous inferences of genetic diversity estimates in large natural populations from finite sample sizes have demonstrated that a sample size of N = 50 field collected female mosquitoes was sufficient to capture most genetic variation and reduce any family effects on results from F_1_ adults [35]. All life-cycle stages of both laboratory colony and wild mosquito populations were maintained under standard insectary conditions (25 ± 2 °C, 80% relative humidity, light:dark cycles of 12:00 h each) and adults were provided with 10% sugar solution.

### Clothianidin bioassays

SumiShield 50WG was tested using WHO susceptibility tests, with minor modifications to the standard guidelines [36]. Whatman^®^ No. 1 filter papers measuring 12 cm by 15 cm were treated with candidate diagnostic doses of SumiShield 50WG (containing 50% clothianidin) diluted in distilled water. A stock solution was prepared by diluting 264 mg SumiShield 50WG in 20 ml distilled water. Two millilitres of the mixed solution was pipetted evenly onto each filter paper and stored at 4 °C until use. Filter paper treated with 2 ml of distilled water was used as the negative control. Exposure time for clothianidin was set at 60 min. Following exposure, mosquitoes were transferred to untreated holding tubes and provided with lightly moistened cotton wool containing 10% sugar solution (changed daily). Knock-down was recorded at 30 and 60 min. Mortality was recorded 1, 2, 3, 4, 5, 6 and 7 days after exposure.

### Chlorfenapyr and other bioassays

CDC bottle bioassays were conducted according to published guidelines [30] and were used to determine the diagnostic doses of chlorfenapyr and assess cross-resistance with other insecticides commonly used for vector control in Ethiopia. Each Wheaton 250 ml bottle and its cap was coated with 1 ml of insecticide solution by rolling and inverting the bottles. In parallel, a control bottle was coated with 1 ml of acetone, following which all bottles were covered with a sheet and left to dry in the dark. Mosquitoes were exposed to chlorfenapyr for 60 min and all other insecticides (bendiocarb, deltamethrin, malathion, permethrin and propoxur) for 30 min. Following exposure, mosquitoes were transferred to a paper cup covered with untreated netting, provided with lightly moistened cotton wool containing 10% sugar solution (changed daily) and monitored at 24 h, 48 h, and 72 h. Knock-down was recorded at 30 min (all other insecticides) or 60 min (chlorfenapyr) and mortality at 24 h, 48 h and 72 h after exposure.

### Diagnostic dose determination

A diagnostic dose is defined as the dose of insecticide that kills 100% of susceptible mosquitoes within a defined period of time [30]. Based on guidance from Sumitomo Corporation and the Africa Indoor Residual Spraying program (AIRS), a diagnostic dose of 2% w/v clothianidin (13.2 mg active ingredient per paper, equivalent to 734 mg ai/m^2^) was recommended (unpublished data). Doses tested for clothianidin in this study were 0.0625 0.125, 0.25, 0.5, 1, and 2%. Previous evaluations conducted by the CDC identified 100 µg/bottle as a putative reference diagnostic dose for chlorfenapyr (unpublished data). In this study, a range of lower and higher doses were tested in bottle bioassays: 0.78125, 1.5625, 3.125, 6.25, 12.5, 25, 50, 100, and 200 µg/bottle.

Bioassays to determine the diagnostic doses of clothianidin and chlorfenapyr were conducted using both DZ and F_1_ generation wild mosquitoes. Multiple batches of 20–25 unfed, 2–5 days old, female mosquitoes were exposed to each dose of insecticide. All bioassays were conducted in temperatures of 27 ± 3 °C with relative humidity of 75–85%.

### Cross-resistance testing

Levels of susceptibility to carbamate (bendiocarb and propoxur), organophosphate (malathion) and pyrethroid (deltamethrin and permethrin) insecticides were assessed among the same population of field-collected mosquitoes. Multiple batches of 20–25 unfed, 2–5 day old F_1_ mosquitoes were exposed for 30 min to technical grade permethrin (21.5 µg/bottle), deltamethrin, bendiocarb and propoxur (all 12.5 µg/bottle) and malathion (50 µg/bottle) in CDC bottle bioassays and knock-down/mortality scored every 15 min for up to 2 h [30]. For bendiocarb, deltamethrin and permethrin, additional bioassays were conducted with two, five and ten times the diagnostic dose [30].

### Data analysis

Generalized linear mixed models were fit to replicate data to estimate the mean mortality proportion when comparing exposed and control female *An. arabiensis* DZ colony mosquitoes to evaluate putative diagnostic doses of clothianidin and wild *An. arabiensis* to test different doses of chlorfenapyr [37]. Random intercepts were included in these models for each batch of mosquitoes tested. These comparisons utilize model-based estimates and post hoc contrast statements to compare exposed and control mosquitoes. In all other evaluations, models did not achieve convergence largely due to a lack of variability between replicates. For these evaluations, replicates are aggregated and evaluated using Pearson’s Chi-squared test or, when the expected number of events in a cell fell below 5, Fisher’s Exact test and odds ratios were calculated using the conditional maximum likelihood. All phenotypic data were interpreted according to the WHO guidelines: mortality of 98% or higher indicates susceptibility, mortality of 90–97% is suggestive of resistance and mortality of less than 90% indicates resistance [36]. Because mortality of control mosquitoes for chlorfenapyr, carbamates, organophosphates and pyrethroids consistently fell below 5%, Abbott’s formula was not required to correct mortality. Given the extended holding period for clothianidin, bioassay data were not discarded if control mortality was greater than 20% by day 3; control data are reported in parallel with test replicates. Analyses were performed in R version 3.5.1 [38], with the level of significance set at α = 0.05.

## Results

### Diagnostic dose determination: clothianidin

**Fig. 1:**
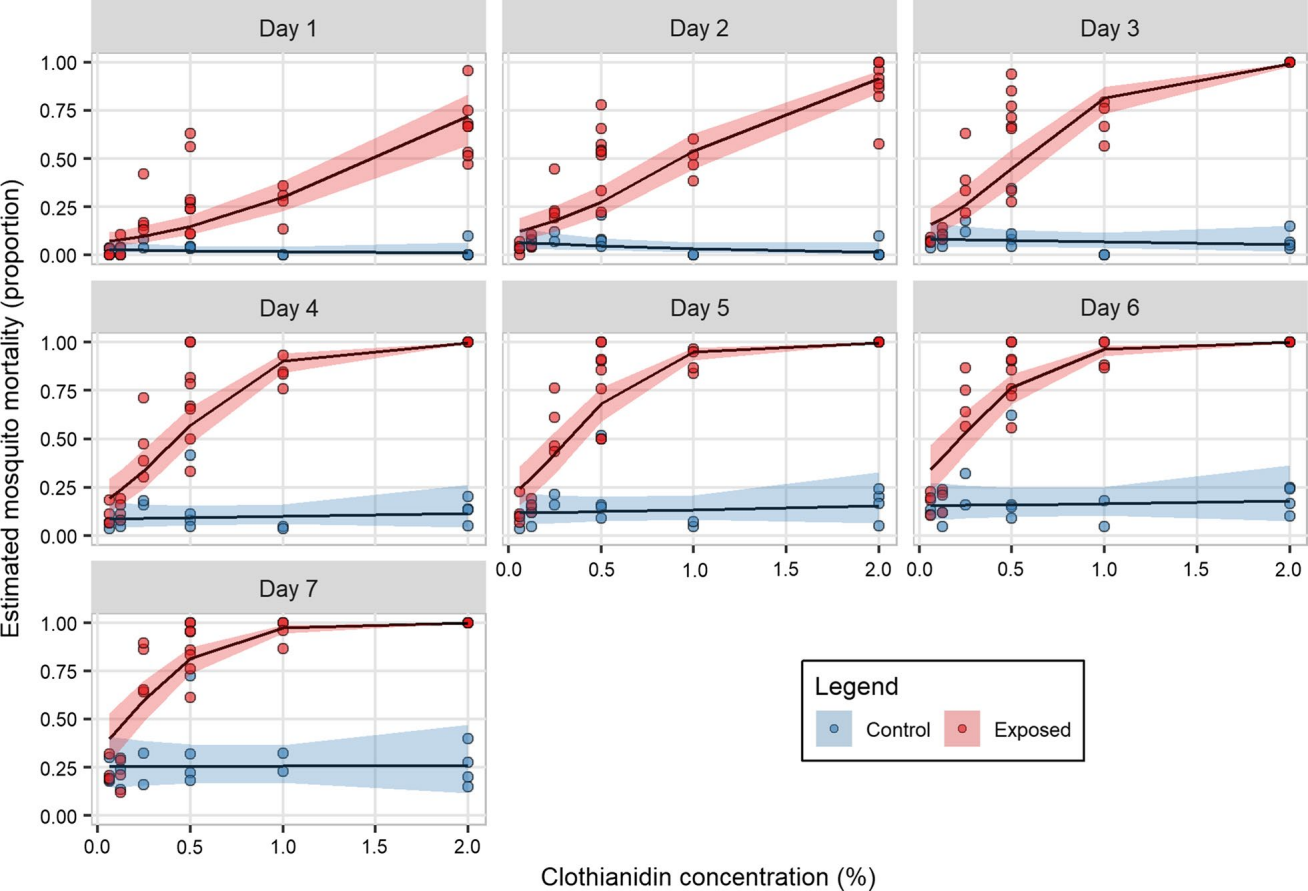
Estimated proportion mortality and 95% confidence intervals from generalized linear mixed models for days 1 through 7 post-exposure of the susceptible DZ *Anopheles arabiensis* strain exposed to six doses of clothianidin or a negative control in WHO susceptibility tests. Doses ranged between 0.0625 and 2%

**Fig. 2:**
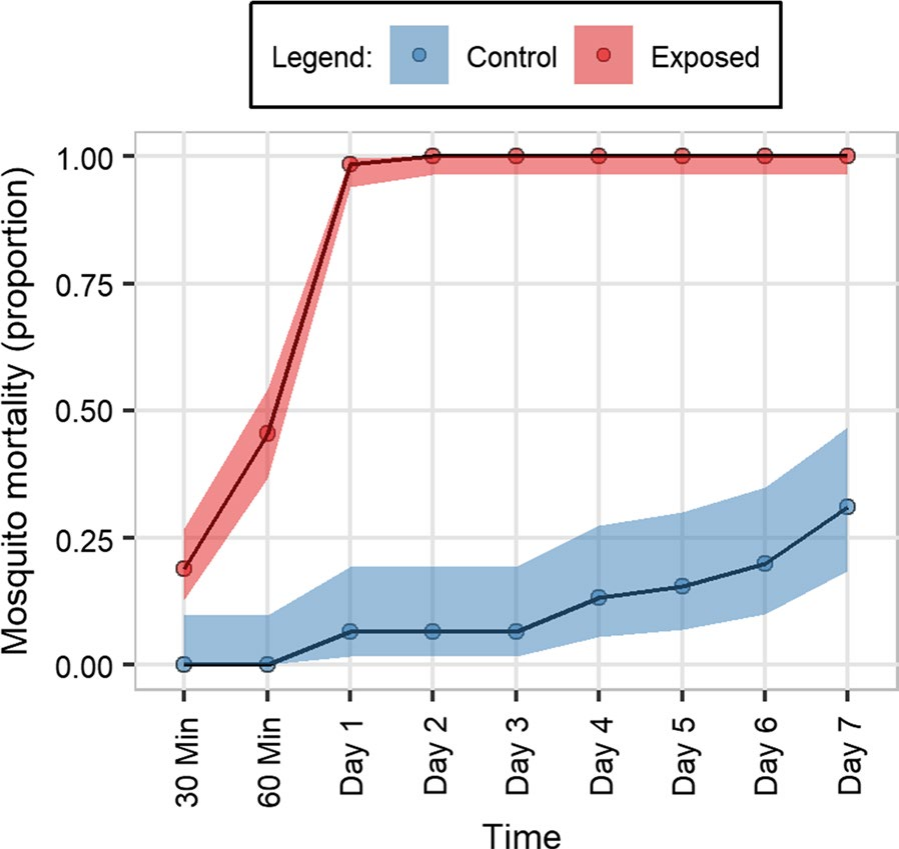
Proportion mortality of wild *Anopheles arabiensis* exposed to 2% clothianidin and controls in WHO susceptibility tests. Knock-down is presented for 30 and 60 min and mortality for days 1–7 post-exposure (with 95% confidence intervals using Wilson’s formula with Yates’ continuity correction)

A total of 1,265 female *An. arabiensis* DZ colony (n = 855 exposed and n = 410 control) (Fig. 1) and 177 wild *An. arabiensis* (n = 132 exposed and n = 45 control) (Fig. 2) mosquitoes were used to evaluate putative diagnostic doses of clothianidin. Complete mosquito mortality was observed at 2% clothianidin in both the wild and laboratory strains at 2 and 3 days post-exposure, respectively. Knock-down at both 30 min (wild: 19%; 25/132 vs. DZ: 1%; 2/200, OR = 23.13, CI 4.38–122.29, p < 0.0001) and 60 min (wild: 45%; 60/132 vs. DZ: 8%; 15/200, OR = 10.28, CI 5.02–21.04, p < 0.0001) was significantly higher in the wild population compared to the susceptible strain. Results from both knock-down rates and time to 100% mortality indicate that the wild population was more susceptible to 2% clothianidin, compared to the laboratory DZ strain.

**Fig. 3:**
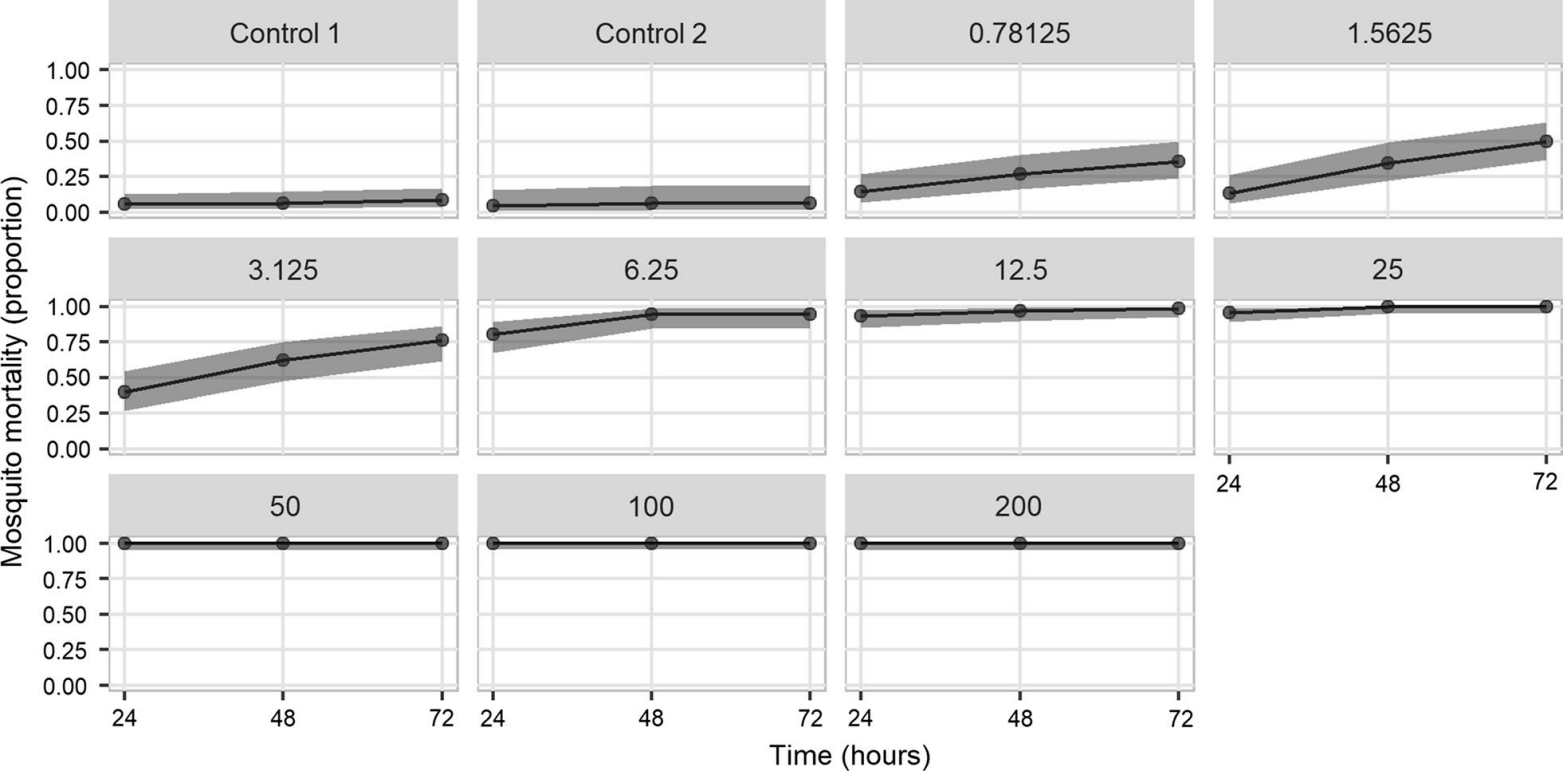
Proportion mortality of the susceptible DZ *Anopheles arabiensis* strain exposed to nine doses of chlorfenapyr using CDC bottle bioassays and control replicates. Doses ranged between 0.78125 and 200 μg/bottle. Mortality is presented for 24, 48 and 72 h post-exposure (with 95% confidence intervals using Wilson’s formula with Yates’ continuity correction)

Apart from 2%, no other tested clothianidin concentration achieved 100% mortality with the DZ strain by the end of the 7-day holding period. As expected, a clear decrease in toxicity was observed as the insecticide concentration was reduced. Mortality to insecticide doses below 0.125% were not significantly different to that of the controls by day 7 (OR = 1.92, CI 0.78–4.71, *p *= 0.16 at 0.0625%). Controls for all tests were maintained for the 7-day holding period, even if test replicates reached 100% mortality before this cut-off. By day 7, the majority of control replicates had a mortality rate > 20%; mean mortality of DZ and wild mosquitoes at day 7 was 31% and 27%, respectively (Figs. 1, 2).

### Diagnostic dose determination: chlorfenapyr

**Fig. 4:**
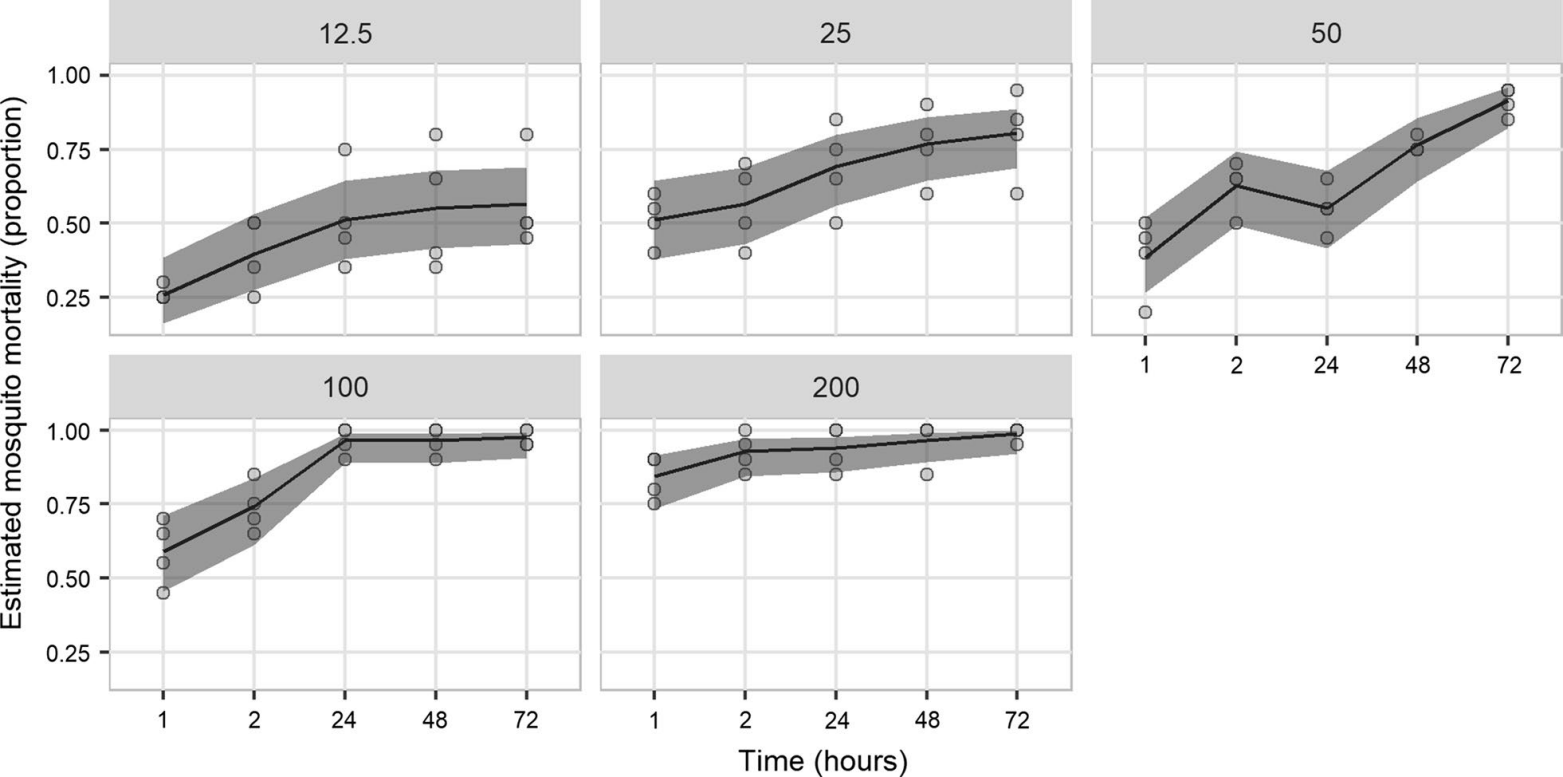
Estimated proportion mortality and 95% confidence intervals from generalized linear mixed models of wild *Anopheles arabiensis* exposed to five doses of chlorfenapyr using CDC bottle bioassays. Doses ranged between 12.5 and 200 μg/bottle. Mortality is presented for 1, 2, 24, 48 and 72 h post-exposure. Control mortality in all assays was 0%

A total of 840 female *An. arabiensis* DZ colony mosquitoes (n = 701 exposed and n = 139 control) (Fig. 3) and 500 wild *An. arabiensis* (n = 400 exposed and n = 100 control) (Fig. 4) were used to test different doses of chlorfenapyr. For the DZ colony strain, the putative diagnostic dose of 100 μg/bottle was confirmed to produce 100% mortality after 24 h. Complete mortality within 24 h was also observed at 50 μg/bottle and for 25 μg/bottle after 48 h, indicating that a lower dose may be equally effective at controlling this laboratory population. A reduction in dose response after 24 h started at 25 μg/bottle (96% mortality; 92/96) and steadily declined with decreasing concentration (Fig. 3). Complete mortality by 72 h was no longer observed at 12.5 μg/bottle (99% mortality; 87/88).

**Fig. 5:**
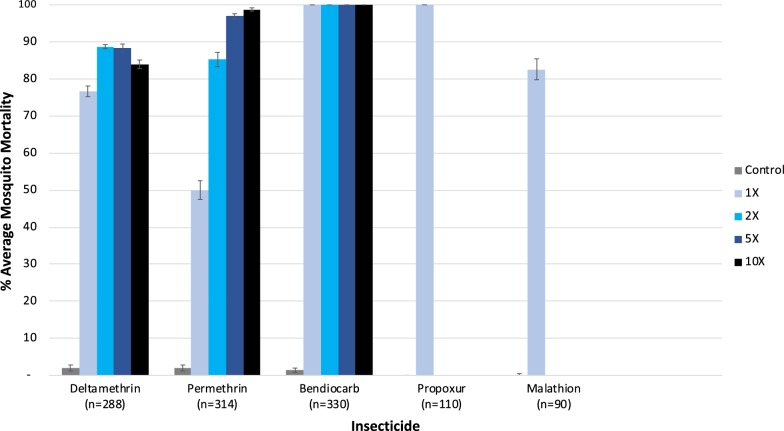
Mean percent mortality of wild *Anopheles arabiensis* exposed to one, two, five or ten times the diagnostic doses of pyrethroids (deltamethrin and permethrin) and the diagnostic doses of carbamates (bendiocarb and propoxur) and organophosphate (malathion) insecticides. Mortality is presented for 30 min post-exposure (with 95% confidence intervals)

By comparison, complete mortality was not observed with wild *An. arabiensis* for any dose within 72 h, suggesting that a higher concentration or longer holding period is required to kill this population. For the recommended diagnostic dose of 100 μg/bottle, mortality peaked at 98% (78/80) after 72 h and the highest rate was observed at 200 μg/bottle after 72 h (99%; 79/80). In comparison to the DZ strain, the wild population produced a more pronounced reduction in response following each subsequent decrease in concentration (Fig. 4). There was no significant difference in mean mosquito mortality between the susceptible and wild strains at 24 h, 48 h, and 72 h for doses of 100 μg/bottle (Fisher’s Exact test, OR = 0, CI 0–1.76, *p *= 0.07; OR = 0, CI 0–1.76, *p *= 0.07; and OR = 0, CI 0–3.89, *p *= 0.18 at 24 h, 48 h and 72 h, respectively) and at 48 h and 72 h for doses of 200 μg/bottle (Fisher’s Exact test, OR = 0, CI 0–1.98, *p *= 0.09; and OR = 0, CI 0–32.17, *p *= 0.45 at 48 h and 72 h, respectively; OR = 0, CI 0–0.88, *p *= 0.02 at 24 h). However, significant differences in survival between the susceptible and wild strains were observed at dose 50 μg/bottle starting at 24 h (*p *< 0.0001).

### Cross-resistance testing

A total of 1132 wild female *An. arabiensis* (955 F_0_ and 177 F_1_ generation) were used to assess susceptibility and cross-resistance to key public health insecticides routinely used in Ethiopia (Fig. 5). Among this wild population, moderate to intense pyrethroid resistance was evident, with proportions of mosquitoes capable of surviving two, five and ten times the diagnostic dose of deltamethrin and permethrin (Fig. 5). In addition, decreased susceptibility to the organophosphate malathion was observed (average mosquito mortality of 83%). No resistance was detected to either carbamate insecticide evaluated (bendiocarb or propoxur); 100% mosquito mortality was achieved within 15 min of exposure. For all bioassays, mortality was recorded for a total of 120 min. For both deltamethrin and permethrin, mosquitoes continued to survive past 30 min of exposure to ten times the diagnostic dose (+15 min, 1/81 alive for permethrin and +45 min, 4/54 alive for deltamethrin) but all replicates reached 100% mortality by 2 h. Similarly, for malathion, all survivors died within 15 min past the exposure period (total 45 min).

## Discussion

Given the limited number of insecticides available for public health use, there is an urgent need to evaluate alternate chemicals with new modes of action to improve the control of resistant vector populations [7]. This study determined the diagnostic doses of clothianidin (neonicotinoid) and chlorfenapyr (pyrrole) for resistance monitoring of field populations of *An. arabiensis* in Ethiopia and also investigated the prevalence of cross-resistance to other chemicals already in use by the NMCP. Diagnostic doses for chlorfenapyr at 100 µg/bottle and clothianidin at 2%/filter paper (both using 60 min exposures) against the susceptible laboratory-reared *An. arabiensis* DZ strain achieved complete mortality by 24 h and 72 h, respectively. These testing conditions are therefore appropriate for this laboratory population and can be used to screen for susceptibility to these compounds in the field, as well as to monitor residual efficacy of interventions longitudinally.

By comparison, field collected *An. arabiensis* exposed to 2%/filter paper clothianidin, demonstrated significantly higher rates of knock-down at 30 and 60 min and reached 100% mortality 24 h earlier than the laboratory DZ strain. It is conceivable that this increased susceptibility may result from a fitness cost or negative cross-resistance incurred by the presence of other resistance traits. A similar phenomenon has been described for the neonicotinoid, dinotefuran, which was more toxic against carbamate-resistant *Culex quinquefasciatus*, compared to a susceptible strain, in both larval bioassays and topical applications to adults [39]. These results were attributed to insensitive acetylcholinesterase being less efficient at degrading nicotinic substrates, such that in the presence of dinotefuran, the concentration of acetylcholine could increase more rapidly at the synaptic level in carbamate resistant individuals, leading to earlier saturation of nicotinic receptors and higher levels of mortality [39]. Additional laboratory studies have reported that an *Anopheles stephensi* colony which was resistant to DDT, malathion and deltamethrin, required lower lethal concentrations of the neonicotinoids imidacloprid, thiacloprid and thiamethoxam, compared to a susceptible counterpart [40], while a tri-mixture of PBO, deltamethrin and dinotefuran was more effective than a combination of PBO and deltamethrin in killing a pyrethroid resistant *An. gambiae* strain (VKPR: homozygous for *kdr*) [26]. In the latter case, it was proposed that PBO blocked mixed-function oxidase (MFO) detoxification of deltamethrin, allowing the rate of miniature excitatory postsynaptic potentials and resulting acetylcholine release to increase, with dinotefuran competitively inhibiting the inactivation of nicotinic receptors.

In the present study, additional testing demonstrated that the wild *An. arabiensis* population had higher tolerance to the organophosphate malathion as well as intense resistance to the pyrethroids deltamethrin and permethrin, with survivors at five and ten times the diagnostic insecticide dose. Complete susceptibility to the carbamates bendiocarb and propoxur was also observed. This field population has previously been characterized by moderate L1014F *kdr* allele frequencies, an absence of the G119S-*Ace*-*1*^*R*^ mutation and partial synergism to pyrethroids following PBO exposure [29, 32, 41]. To date, the underlying metabolic mechanisms conferring resistance to organophosphates and pyrethroids await elucidation but the putative negative cross-resistance to clothianidin render this insecticide a promising candidate for inclusion in local vector control campaigns.

The efficacy of chlorfenapyr against resistant mosquito species has been reported from multiple settings [8, 10, 11, 15–18, 32, 42–47]. Because the mode of action for chlorfenapyr differs from that of other neurotoxic insecticides used for malaria vector control, it is anticipated that there is minimal risk for cross-resistance to evolve from currently known metabolic resistance pathways [18]. Furthermore, laboratory studies have demonstrated that PBO can act as an antagonist with chlorfenapyr, reducing mosquito mortality, owing to the involvement of MFOs in the initial conversion step of the pro-insecticide to its active toxic form [11, 48, 49]. This property highlights the potential of this insecticide to manage resistant populations characterized by elevated oxidases, one of the predominant mechanisms conferring insecticide resistance across sub-Saharan Africa [50]. However, in this study, complete mortality was not achieved with the wild *An. arabiensis* population at any dose within 72 h indicating that a higher concentration or longer holding period may be required. Previous laboratory evaluations have highlighted temperature and time of bioassay, as two factors which can influence mortality; activation of chlorfenapyr and disruption of respiratory pathways is enhanced when the mosquito is more metabolically and behaviourally active [51]. All of the current testing was conducted during the daytime, at constant, regulated temperatures, which may account for lower levels of lethality; in this dataset it is not possible to ascribe incomplete mortality to this scenario or the presence of a small proportion of more tolerant individuals. Study findings reinforce the need to adapt standardized laboratory testing guidelines to take into account the idiosyncrasies of non-neurotoxic insecticides [44, 51].

During testing, a number of limitations were encountered and areas of improvement were identified. Due to issues of crystallization and achieving uniform coating of Wheaton bottles with technical grade clothianidin diluted in acetone, formulated SumiShield 50WG was used to treat filter papers for WHO susceptibility tests. In addition to introducing batch variability between ‘home-made’ filter papers, other additives contained within this formulation may have also contributed an unmeasurable degree toward mosquito mortality. In future studies, it may be appropriate to evaluate alternate methodologies and solvents to facilitate the use of technical grade insecticide in bioassays. In the clothianidin bioassays, maintaining control mosquitoes for 7 days presented issues, with mortality exceeding 20% by day 5 in a number of replicates, possibly because of old age or starvation due to a lack of blood meal. Given that complete mortality in both wild and field populations was achieved within 3 days, longer holding periods may not be needed for future testing of these particular populations. The clothianidin results have been interpreted relative to reports of other neonicotinoids; however, observations may not be generalizable as each compound is known to vary in binding potency to the nAChR receptor and may, therefore, exert distinct effects between insect species [21]. Unfortunately, due to insufficient sampling, the wild *An. arabiensis* used in chlorfenapyr testing was not collected at the same time as those used to assess the diagnostic doses of clothianidin or other insecticides, preventing complete comparisons between these groups. However, the resistance profiles of this population have been shown to be consistent over the last couple of years [29]. Finally, levels of insecticide resistance of wild Ethiopian mosquitoes are highly focal and heterogenous across the country [29, 41] and ideally such screening (including different concentrations and exposure times) and cross-resistance testing would be extended to other field populations with different resistance intensities and underlying molecular and metabolic mechanisms. Future studies may also consider including synergists to explore possible metabolic mechanisms that can explain the observed cross-resistance, especially for clothianidin which is known to have a synergistic effect with PBO [22, 26]. This work only examined lethal effects of exposure, while some neonicotinoids are known to modify insect behaviour at sublethal concentrations; if these compounds impact feeding behaviour, fecundity and/or egg hatchability, these could also be important contributors to reducing vectorial capacity. The range of sub-lethal concentrations identified herein provide potential starting points for such studies.

## Conclusions

This study demonstrated that the putative diagnostic doses of clothianidin and chlorfenapyr are appropriate for monitoring resistance in *An. arabiensis* from Ethiopia. The unique slower acting modes of action of clothianidin and chlorfenapyr have been predicted to impose less selection pressure for the evolution of insecticide resistance compared to faster acting chemicals [52]. Coupled with the absence of cross-resistance (and potential occurrence of negative cross-resistance) to insecticides in current use for malaria control in Ethiopia, vector control interventions incorporating clothianidin and chlorfenapyr warrant consideration for inclusion in the National Malaria Control Programme resistance management strategy, particularly in areas with high pre-existing or emergent resistance to multiple insecticide classes.
